# Fabrication and Characterization of Simple Structure Fluidic-Based Memristor for Immunosensing of NS1 Protein Application

**DOI:** 10.3390/bios10100143

**Published:** 2020-10-16

**Authors:** Nor Shahanim Mohamad Hadis, Asrulnizam Abd Manaf, Mohamad Faizal Abd Rahman, Siti Hawa Ngalim, Thean Hock Tang, Marimuthu Citartan, Aziah Ismail, Sukreen Hana Herman

**Affiliations:** 1 Collaborative Microelectronic Design Excellence Centre (CEDEC), Sains@USM, Universiti Sains Malaysia, Pulau Pinang 11900, Malaysia; 2 Faculty of Electrical Engineering, Universiti Teknologi MARA Cawangan Pulau Pinang, Kampus Permatang Pauh, Pulau Pinang 13500, Malaysia; faizal635@uitm.edu.my; 3 Regenerative Medicine Cluster, Advanced Medical & Dental Institute (AMDI), Universiti Sains Malaysia, Pulau Pinang 13200, Malaysia; siti.hawa.ngalim@usm.my; 4 Infectomics Cluster, Advanced Medical & Dental Institute (AMDI), Universiti Sains Malaysia, Pulau Pinang 13200, Malaysia; tangth@usm.my (T.H.T.); citartan@usm.my (M.C.); 5 Institute for Research in Molecular Medicine (INFORMM), Universiti Sains Malaysia, Kelantan 16150, Malaysia; aziahismail@usm.my; 6 Nano-Electronics Centre (NET), Universiti Teknologi MARA, Shah Alam, Selangor 40450, Malaysia; hana1617@uitm.edu.my

**Keywords:** current-voltage measurement, fluidic-based memristor sensor, NS1 protein, off-on resistance ratio

## Abstract

Non-structural protein 1 (NS1 protein) is becoming a commonplace biomarker for the diagnostic of early detection of dengue. In this study, we sought to use a label-free approach of detecting NS1 protein by harnessing fluidic-based memristor sensor. The sensor was fabricated using sol-gel spin coating technique, by which TiO_2_ thin film is coated on the surface of Indium tin oxide (ITO) and a glass substrate. The sensor was then functionalized with glycidoxypropyl-trimethoxysilane (GPTS), acting as antibody for NS1. The addition of the target NS1 formed an antibody-antigen complex which altered the physical and electrical properties in sensing region. Sensing of the sensor is incumbent upon the measurement of Off-On resistance ratio. Imaging with Field Emission Scanning Electron Microscope (FESEM) evinced the successful immobilization of the antibody and the subsequent capture of the NS1 protein by the immobilized antibody. The detection limit actualized by the developed sensor was 52 nM and the diameter of 2 mm gives the most optimal measurement. The developed sensor demonstrated an immense potential towards the development of label-free diagnostic of early dengue infection.

## 1. Introduction

Memristor is a relatively new device envisioned by Leon O. Chua through his mathematical modelling published in 1971 [1]. In 2008, a group of researchers from Hewlett Packard, led by Dmitri B. Strukov developed the first memristor device made from titanium dioxide (TiO_2_) [2]. Strukov’s paper ever since has become the most referred article in memristor development. Memristor was also deployed in a number of applications including memory [3,4,5,6,7], computing [8] and bio-sensing [9,10,11,12,13,14]. For the bio-sensing application, memristor-based sensor offers dual functions, which can be performed as a sensor and memory at the same time. Therefore, the size of the sensing system when integrated with interface circuit could be reduced by minimizing the number of transistor toward monolithic chip.

Sensing application of memristor was introduced by Carrara et al. in 2012. Several silicon nano-wire memristor bio-sensors were developed for biomolecular detection under dry condition [9,13]. Under dry condition, subsequent to the addition of capturing antibodies and the corresponding targets, the sensors are dried prior to characterization or the measurement of the biomolecular interactions [9,13]. However, the drying process, leads to a longer period of time for the completion of the entire immunosensing process.

Therefore, the need for a faster immunosensing process is crucial for rapid detection, which is beneficial for real-time detection for in situ measurements. Prior to that, the development a fluid-based sensing platform was proposed, whereby the drying process was completely averted. Capitalizing the sensing excellency of memristor, we endeavored to develop an immunosensor for the NS1 protein of dengue virus detection. Dengue virus is the etiological agent of dengue fever. One of the non-structural proteins, NS1, is known to induce strong humoral response and is present at high concentrations in the blood up to nine days after primary and secondary infections [15]. Possessing a very high diagnostic value, NS1 protein was successfully applied in enzyme-linked immunosorbent assay (ELISA)-based tests with the sensitivity scored of 94.1%, 100.0%, and 80.0% for Panbio test, InBios test, and Bio-Rad test, respectively.

The development of the memristor-based sensor is viewed as one of the endeavors to pursue a label-free diagnostic detection of dengue. In light of the diagnostic splendor of the NS1 protein, the sensor developed can be useful for the diagnostic of early stage dengue infection.

## 2. Sensing Principles

**Figure 1:**
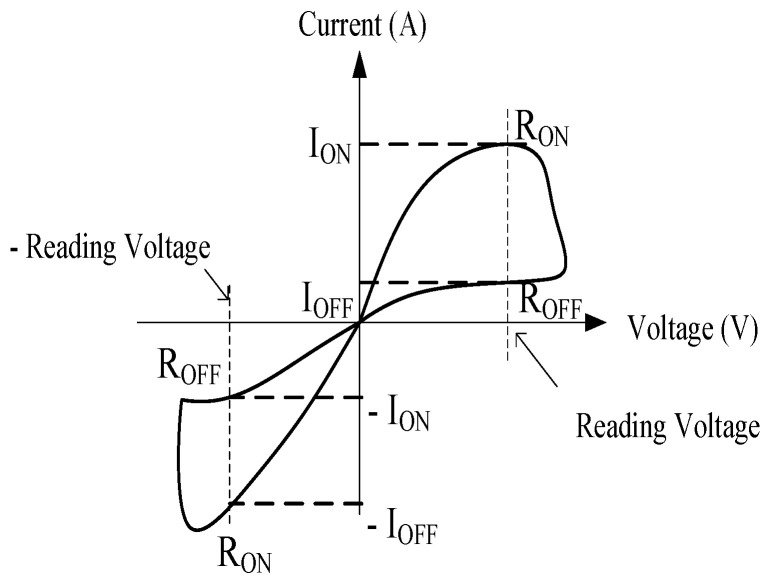
Standard *I-V* curve of a memristor device in a form of pinched hysteresis with ‘ON’ current and ‘OFF’ current position.

The proposed sensor is working based on the Off-On resistance ratio (*R_off_/R_on_*) measurement of the device obtained from current-voltage (*I-V*) curve. This sensing technique manipulates the hysteresis characteristics of *I-V* curve which gives different value of current during forward and reverse voltage ramp. Figure 1 shows the relationship between voltage and current as well as the Off-On resistances.

For immunosensing application, the biological reaction between antibodies and antigen alters the electrical properties of the sensing area (immunosensing platform). The application of voltage across the immunosensing platform causes some current to flow prior to the formation of antibody-antigen complex. In relation to these *I-V* relationship, the electrical resistance, denoted as *R_on_* and *R_off_* can be obtained. The ratio of these resistance, *R_off_/R_on_* taken at specific reading voltage provides useful information that corresponds to the concentration of the formed complex.

Due to hysteresis phenomenon, different pattern of *I-V* curve is normally observed during forward and reverse voltage ramp. In this type of sensing technique, a large hysteresis loop is preferable for better detection performance.

## 3. Methodology

In order to achieve the main objective, the work is divided into three main stages, i.e., sensor development, testing and characterization and performance evaluation.

### 3.1. Sensor Development

In this stage, the development of the proposed sensing device is achieved through the integration of microfluidic chamber and sensing platform. The steps are outlined as follows.

#### 3.1.1. Microfludic Chamber

**Figure 2:**
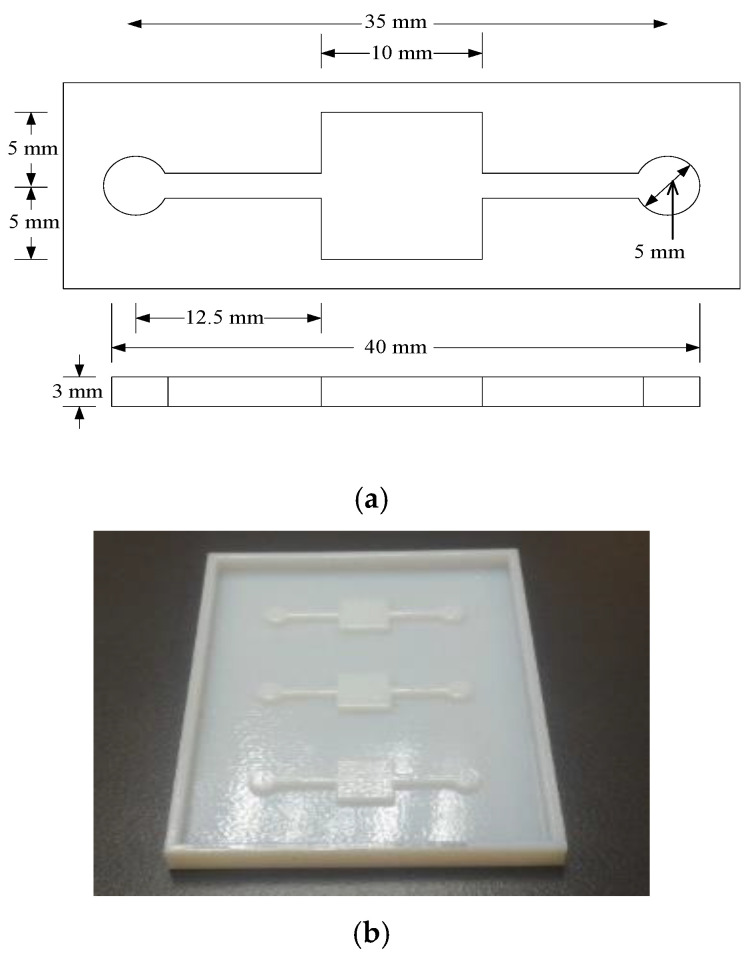

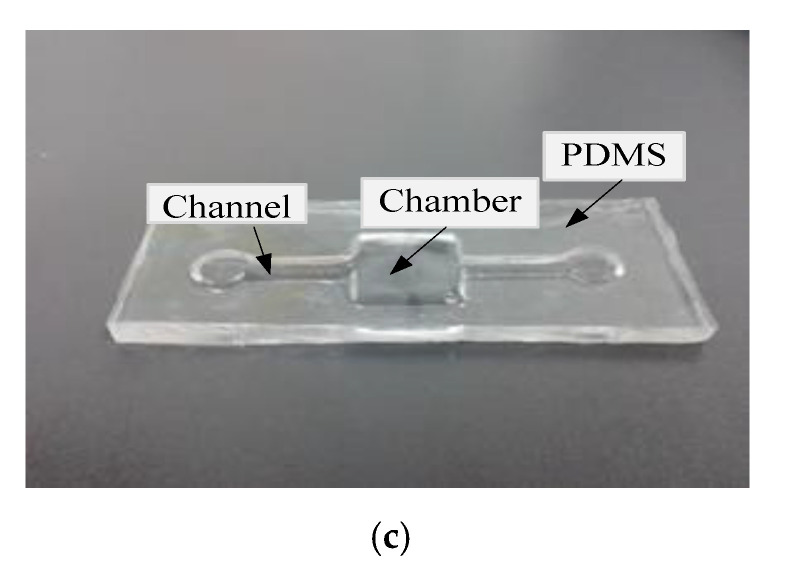
Mold for microfluidic chamber; (**a**) Structural dimension; (**b**) Rapid prototyping mold; (**c**) microfluidic chamber.

Microfluidic chamber is used as a medium for sample and immunoassay transportation as well as to prevent the sample from evaporation process. The structure also guards the sample from any external influences or disturbances that can affect the sensing performance. The fabrication process began by creating a mold to replicate the Polydimethylsiloxane (PDMS) chip. Figure 2a shows the top view design of the proposed structure, having one inlet and outlet, a fluidic-channel and a reaction chamber. By using rapid prototyping (RP) technique, the mold is fabricated as depicted in Figure 2b. RP is a type of freeform fabrication technique, whereby the mold is directly fabricated from computer aided design (CAD) model. By following a standard soflitography process, the PDMS chip is replicated as shown in Figure 2c.

#### 3.1.2. Sensing Platform

**Figure 3:**
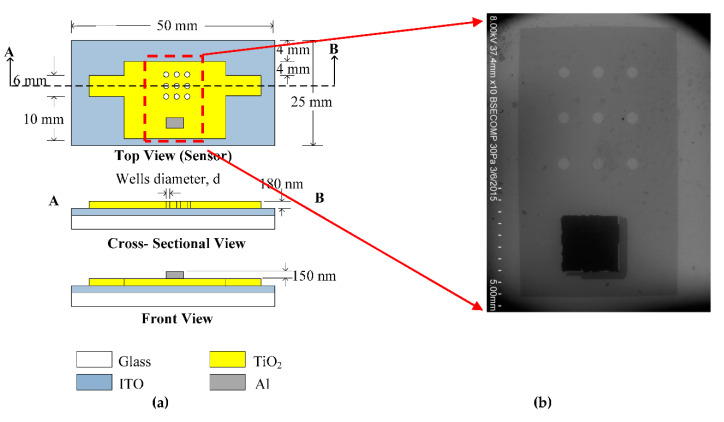
Schematic diagram of sensing platform; and (**a**) image of fabricated sensor (**b**).

Sensing platform provides a platform for bioreaction, signal generation and detection. Figure 3a shows the design of the sensing platform with nine wells in order to maximise the sensing area between antibody and antigen. Figure 3b shows the photograph of fabricated sensor.

**Figure 4:**
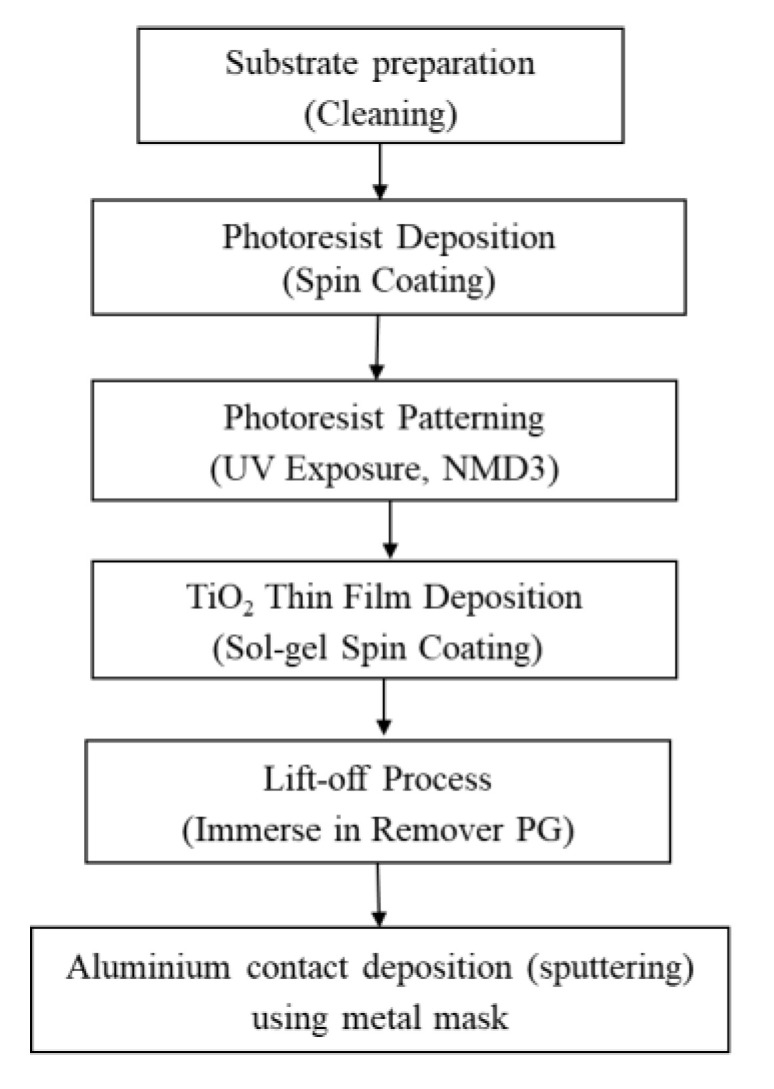
Graphical view of PDMS attachment process using adhesive bonding technique.

In order to realise the structure, microfabrication technique is used. Figure 4 illustrate the fabrication process flow to realise the sensing platform.

Initially, Indium tin oxide (ITO) coated glass substrate (sheet resistance 10~15 ohm/sq) is selected as the platform of the memristor sensor. The ITO coated glass substrate was cleaned using methanol, acetone and distilled water in ultrasonic bath. The substrate was dried using nitrogen gas and baked for 5 min at 160 °C.

Then, LOR (lift-off resist) 30B (Tokyo Ohka Kogyo, Kawasaki, Japan) was deposited on top of the ITO layer using spin coater at 2000 rpm for 25 min. The substrate was pre-baked at 100 °C for 5 min followed by OAP (Tokyo Ohka Kogyo) coating using spin coating technique at 2000 rpm for 25 s. Another layer of positive photoresist type IP3100Hs (Tokyo Ohka Kogyo, Kawasaki, Japan) was also spin-coated on top the of the OAP layer at 2000 rpm for 25 s. The substrate was pre-baked again at 100 °C for 90 s.

The top surface of the substrate is then exposed to ultraviolet light (wavelength, λ = 365 nm) using UV exposure system through a film mask for the photoresist patterning.

Then, the sol-gel TiO_2_ was spin-coated onto the substrate at two speeds; 500 rpm for 5 s and 2000 rpm for 20 s. The sol gel is prepared by mixing two solutions, a mixture of 85.2% ethanol (Prolabo, Selangor, Malaysia), 9.2% glacial acetic acid (Hmbg, Hamburg, Germany) and 5.6% titanium isopropoxide (Aldrich, MO, USA) and a mixture of 99% ethanol (Prolabo) and 1% Triton X-100 (Sigma Aldrich), which are prepared separately. The deposited thin film device was then post-baked at 200 °C for 10 min.

Next, the lift-off process was done by dipping the substrate into Remover PG (Microchem, TX, USA) overnight.

The last process in sensing platform development is achieved through aluminum contact deposition which was performed using direct current (dc) sputtering technique. The target used for sputtering technique was high in purity of 99.99% Al target. The sputtering parameters were as follows: DC power, room temperature, Ar gas of 18 sscm, a base vacuum pressure of 2.02 × 10^−5^ mbar and a process vacuum pressure of 6.02 × 10^−3^ mbar.

#### 3.1.3. Sensor Bonding

**Figure 5:**
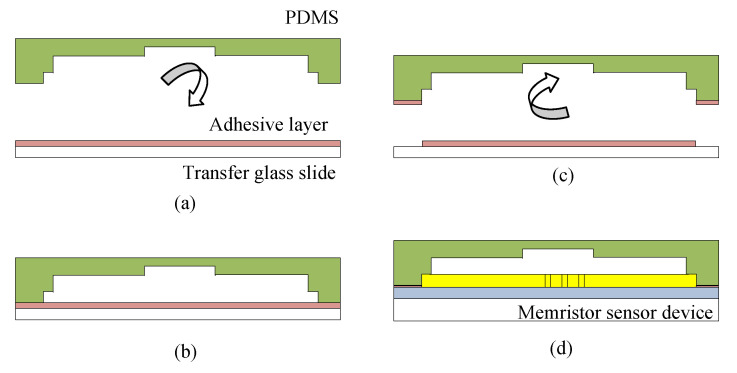
Graphical view of PDMS attachment process using adhesive bonding technique (**a**) Uniform adhesive layer on transfer glass; (**b**) adhesion process; (**c**) lift off with adhesive layer at bonding contact point; (**d**) bonding process (PDMS chamber attached on sensing platform).

In order to complete the final stage of device development, the PDMS chamber is attached on top of the sensor surface by using adhesive bonding technique. By this technique, PDMS is placed on a transfer glass slide which was spin coated with ultraviolet (UV) curable adhesive layer for 3 s. The PDMS was then lifted off and attach permanently onto the memristor sensor device. The technique is illustrated in Figure 5. This technique provides a uniform layer of adhesive to the PDMS contact and thus provide a well implemented fluidic-based memristor sensor.

### 3.2. Testing and Characterisations

In order to characterise the proposed sensor, suitable techniques are selected to determine the sensing capability for performance evaluation process. For that purpose, current-voltage (*I-V*) measurement and field emission scanning electron microscope (FESEM) are used once the samples have been properly prepared.

#### 3.2.1. Surface Functionalisation and Sample Preparation

Prior to device testing, the surface of the sensing area is functionalized with a suitable antibody, in order to promote the formation of complex when reacting with the antigen. In this work, the TiO_2_ thin film surface on Indium tin oxide (ITO) coated glass substrate sensor is functionalized with GPTS (glycidoxypropyl-trimethoxysilane) prior to immobilization with amine-containing molecules. The sensor was first cleaned using plasma cleaner to remove impurities and contaminants [16]. The sensor surface was then incubated in ethanol containing 10 mM acetic acid and 1% GPTS for one hour [9,13]. The surface was then dried under nitrogen stream and baked at 110 °C for 15 min [9,13].

The sensor was washed using phosphate-buffered saline (PBS) solution and immobilized with PBS containing 0.05 mg/mL of anti-DENV-1 NS1 glycoprotein monoclonal antibody. The sensor was then incubated at room temperature for 3 h in a humid chamber before washing three times with PBS. The remaining active GPTS was blocked by 10 mM ethanolamine for 1 h [9,13,17] followed by washing process using PBS. The free spaces between antibodies were blocked by incubating the sensor surface with 3% gelatin (Sigma) in PBS (*v*/*v*) for 30 min [9,13]. The sensor was rinsed once using PBS and dried using nitrogen gas.

Once the sensing area has been functionalized, different concentrations of DENV-1 NS1 glycoprotein (full length, 48 kDa) of 52 nM, 104 nM, 208 nM and 416 nM were added onto the antibody-functionalized sensor before washing with PBS.

#### 3.2.2. FESEM

**Figure 6:**
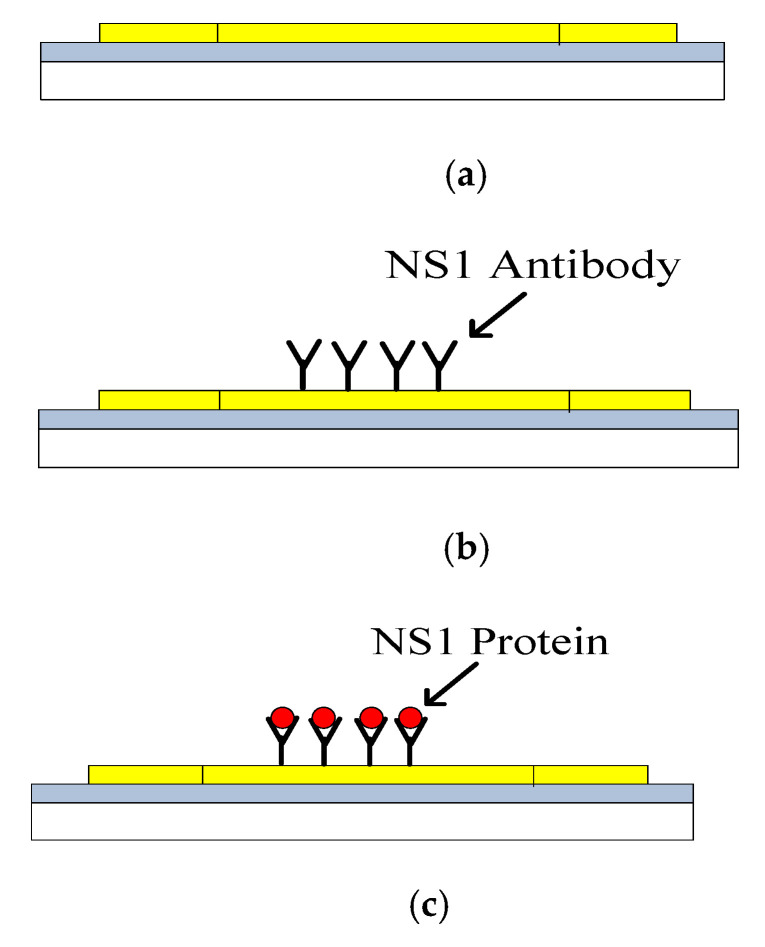
Three phases of characterisation samples; (**a**) Phase I: Free sensor surface; (**b**) Phase II: Functionalised sensing area with antibody; (**c**) Phase III: Sensor surface with antibody-antigen complex formation.

The images of antibody-antigen complex formation was also taken based on Field Emission Scanning Electron Microscope (FESEM). The FESEM unit used in this study is Ultra-High Resolution (UHR-FESEM) model FEI Nova NanoSEM 450. The morphology of the sensing area (TiO_2_) observed for three different phases. i.e., Phase (I): free surface of the TiO_2_, Phase (II): Surface of the TiO_2_ functionalised with NS1 antibody and Phase (III): NS1 protein complexed with the antibody immobilized surface of the TiO_2_. The concept is depicted in Figure 6.

#### 3.2.3. *I-V* Curve

**Figure 7:**
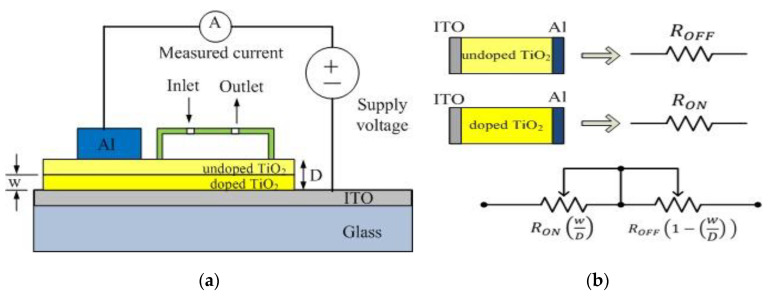
Current-Voltage behavior for memristor mechanism (**a**) TiO_2_ layer and (**b**) equivalent circuitry.

The sensing principle of the sensor is based on memristor model by Miller et al., 2010 [18] and Strukov et al., 2008 [2]. The TiO_2_ thin film is fabricated with perfect layer of TiO_2_ and TiO_2_ layer with oxygen vacancies as shown in Figure 7a. As shown in the Figure 7b, the undoped layer is represented by a resistor with resistance value of off resistance (*R_OFF_*), and the doped layer is represented by a resistor with value of on resistance (*R_ON_*). The simplified equivalent circuit for the memristor device is in series connection between *R_ON_* and *R_OFF_* with the *R_ON_* and *R_OFF_* depends on TiO_2_ thin film thickness, *D* and the thickness of doped layer, *w* [2]. In this paper, Aluminium and ITO were selected as an electrode. Theoretically, the thickness of the switching medium TiO_2_ is constant described by *D*. The thickness of the switching medium that is saturated by oxygen vacancies, which assist conduction is described by the function *w*. If a positive voltage is applied to the doped side, the vacancies being positively charged will be repelled and drift into the undoped region restricted by the mobility of the oxygen vacancies given by *μ_v_* as shown in Equations (1)–(3). Strukov et al. developed memristor model for the memristor device and the relationship between voltage and current represent by Equation (1) [2]. This equation developed for the simplest case of ohmic electronic conduction and linear ionic drift in a uniform field with average ion mobility, *µ_V_*. Eventually *w* will become equal to *D* resulting in the ON state as shown 7b. If the bias voltage is swapped, the oxygen vacancies will recede. Eventually *w* will be equal to 0 resulting in the OFF state as the vacancies are completely pushed to one side as shown in Figure 7b. The changing of *w* due to supply voltage will be causing the change of resistance for *I-V* characteristic of memristor behavior. Based on the principle, the resistance of memristor will change when the immobilized antibody was bonded with antigen (NS1 protein) on TiO_2_ due to changing of undoped region. Then, it will change the behavior of *I-V* curve hysteresis. Large loop size will produce large value off-on resistance ratio and vice versa.

(1) $$\frac{dw\left(t\right)}{dt}=\mu_{V}\frac{R_{ON}}{D}i\left(t\right)$$

(2) $$w\left(t\right)=\mu_{V}\frac{R_{ON}}{D}q\left(t\right)$$

(3) $$M\left(q\right)=R_{OFF}\left(\frac{\mu_{V}R_{ON}}{D^{2}}q\left(t\right)\right)$$

**Figure 8:**
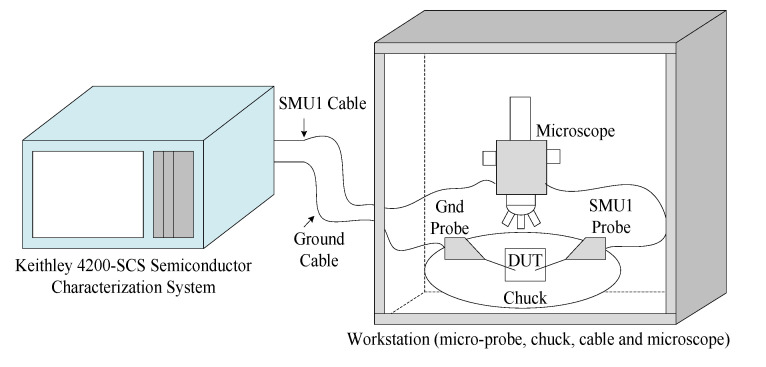
Illustration of Hysteresis *I-V* characterization setup for memristor behavior.

**Figure 9:**
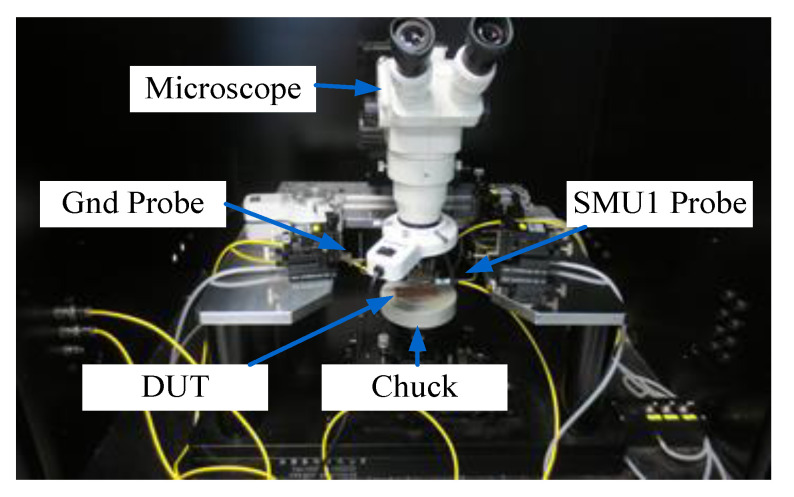
Photograph image of Hysteresis *I-V* characterization setup for memristor behavior.

The hysteresis loop *I-V* curve obtained during the *I-V* measurement which formed by sweeping voltages from 0 V to +1 V, followed by +1 V to −1 V, and the last sweep was from −1 V to 0 V. This process was performed using Keithley 4200–Semiconductor Characterization System (SCS). The schematic diagram and image of the measurement setup, which comprise the characterization system and a workstation, is shown in Figure 8 and Figure 9. The workstation consists of a chuck, micro-probe, cable, microscope, and others. The memristor sensor was placed on the chuck, and the micro probe was positioned on the top and bottom contacts of the sensor device. An SMU1 probe was placed on the top contact, and a Gnd probe was placed on the bottom contact.

For this study, the sensing principles of this proposed sensor is based on the Off-On resistance ratio measurement of the device which is obtained from *I-V* curve. *I-V* curve for a normal memristor sensor is in pinched hysteresis shape which presents a loop at positive and negative reading voltage (Figure 1). The ratio represents the loop size of the *I-V* curve, which is changing in accordance to the behaviour of the liquid used [19]. *I-V* curve for phase III was obtained after 30 min of incubation at room temperature. The Off-On resistance ratio, *R_r_* was then determined using Equation (4),

(4) Rr=[VrIOFFVrION]

where, *Vr* is the reading voltage, *I_off_* is the current at a selected reading voltage during negative voltage ramp, and *I_on_* is the current at a selected reading voltage during positive voltage ramp.

### 3.3. Performance Measurements

The sensitivity of the sensor is used to determine the minimal NS1 protein able to be detected by the sensor. The sensitivity measurement was also used to determine the most optimal diameter size of the wells on the sensor. Four different diameters are selected as 0.5, 1.0, 1.5 and 2.0 mm. From the plot of Off-On resistance ratio against concentration, two points were selected, ratio at point 1 (*Rat* 1) and point 2 (*Rat* 2). The points correspond to two selected concentrations, concentration 1 (C*onc* 1) and concentration 2 (*Conc* 2) respectively. The sensitivity, *S* for this memristor sensor is then determined by using Equation (5),

(5) $$S_{}=\left[\frac{Rat\;1-Rat\;2}{Conc\;1-Conc\;2}\right]$$

where, *Rat* 1 and *Rat* 2 are the Off-On resistance ratio at two selected points and *Conc* 1 and *Conc* 2 are the concentration of the target samples at these points. These concentrations represent the concentration of NS1 protein that have been prepared during testing.

The well diameter that produces the largest change of Off-On resistance ratio between two samples with different concentrations indicate the most optimal diameter with the best sensitivity.

## 4. Results and Discussions

In this work, a label-free strategy of detecting NS1 protein was developed. The Indium tin oxide (ITO) coated glass substrate sensor is coated with TiO_2_ thin film, which is then functionalized with GPTS. Amine containing antibodies interact with the epoxide functional group containing GPTS, resulting in the immobilization of the antibodies on the surface of the platform [20]. Anti-NS1 antibodies immobilized on the surface of the sensor platform reacts with NS1 protein, resulting in the antibody-protein complex.

A complete device with PDMS chamber attached on top of the sensor is developed. The structure is capable of preventing sample evaporation due to the liquid based measurement. Liquid-based measurement is prone to sample evaporation if the sensing surface is not fully covered, especially involving small volume of liquid (microliter). The issue of liquid evaporation was raised by Ettinger and Wittmann, whereby they suggested the usage of a sealed chamber to avoid liquid evaporation [21]. The evaporation will change the behaviour of the liquid and thus produce an inaccurate output. The results obtained from characterisation works are discussed based on the image taken from FESEM and *I-V* curve analysis.

### 4.1. Surface Morphology

**Figure 10:**
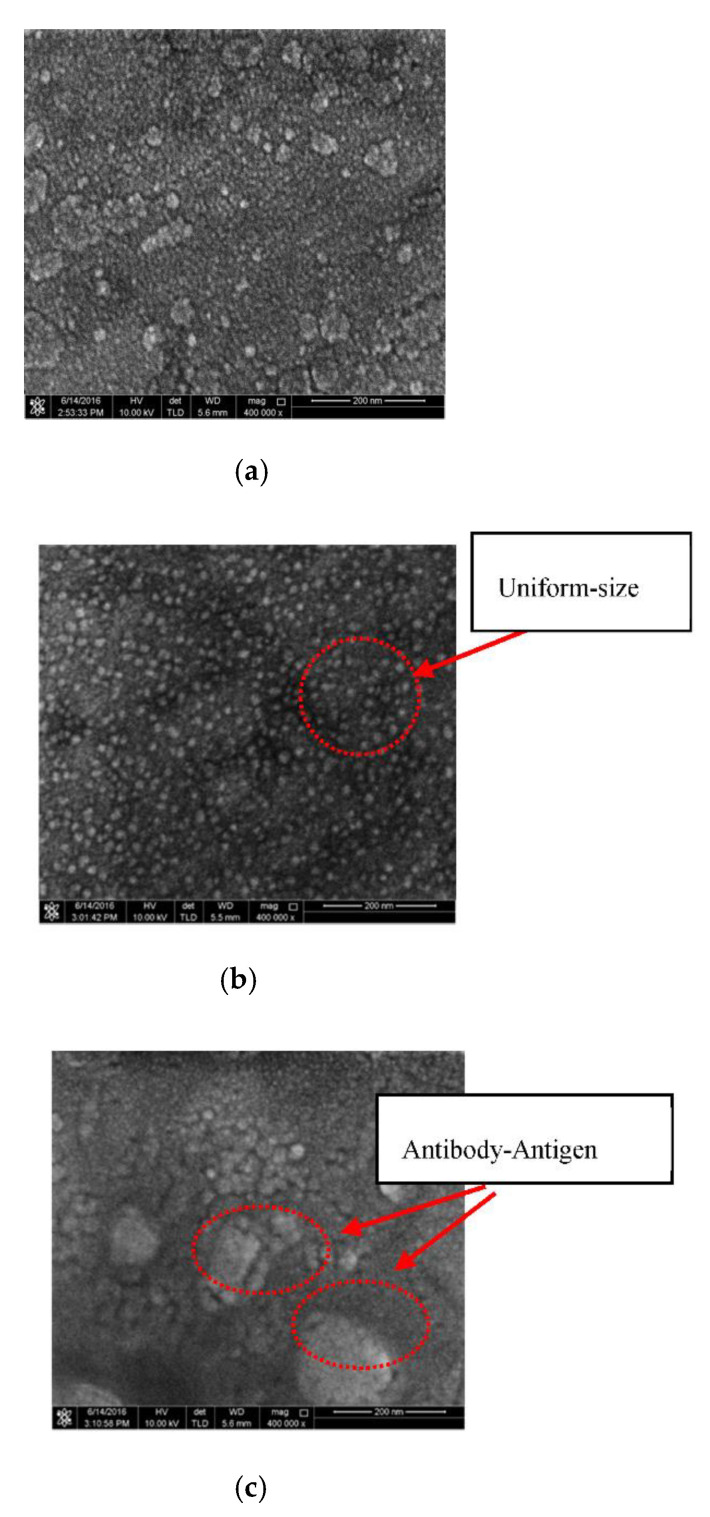
Top view of FESEM image of; (**a**) free sensor (Phase I); (**b**) sensor modified with NS1 antibody (Phase II); and (**c**) modified-antibody sensor with NS1 protein.

The surface morphology of the sensor was studied using FESEM technique. Figure 10 shows the FESEM images captured at the magnification of 400,000 times for three different phases, i.e., phase I: free sensor surface, phase II: functionalized/immobilized sensor surface with NS1 antibody and phase III: sensor surface with antibody-antigen complex formation. The observation region was focused on the surface of TiO_2_ surface, in which the imaging of the complete bio-detection process is performed.

It can be observed from Figure 10b that the NS1 antibody-conjugated sensor surface shows the existence of small particles or white sports, which is absent on the surface-free sensor, as shown in Figure 10a. These white sports represent the NS1 antibodies that were immobilized on the TiO_2_ surface. At 400k magnification level, the surface for TiO_2_ surface with dengue antibody seems filled with small particles or white sports with size of ~10nm diameter, which represent the dengue antibody that has been conjugated on the TiO_2_ surface as reported by Camara et al., 2013 [22]. The modified surface that has been applied with dengue virus presented an obvious change in surface morphology. The surface roughness increases due to dengue virus that captured by the conjugated antibody. The dengue virus has almost similar structure with crystal structure of dengue type 1 envelope dengue protein reported by Nayak et al., 2009 [23]. Subsequently, the complex of antibody-NS1 protein showed an obvious change in surface morphology, as shown in Figure 10c. The surface morphology changes are due to the target NS1 protein captured by the conjugated antibody. The aggregation formation as observed in Figure 10c suggested the formation of hexamer, which is the conformation adopted by the NS1 protein when the protein is secreted during dengue infection [24]. Although, Figure 10a also exhibits white spots, the white spots are not as large as in Figure 10c. The white spots represent large aggregates that arise from the formation of the antibody-NS1 hexamer complex. These images thus confirmed the biological reaction that occurs on the surface of the sensor after being functionalized with suitable antibody.

### 4.2. Hysteresis Loop Size and Resistance Ratio

**Figure 11:**
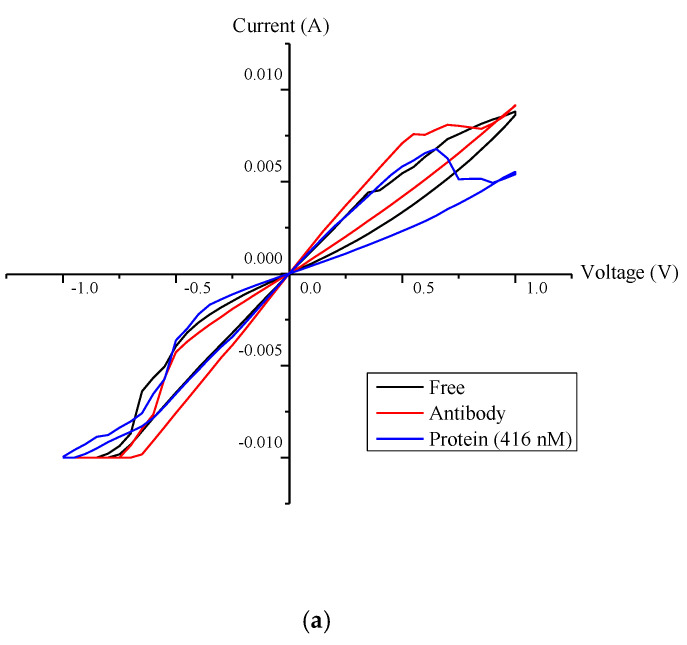

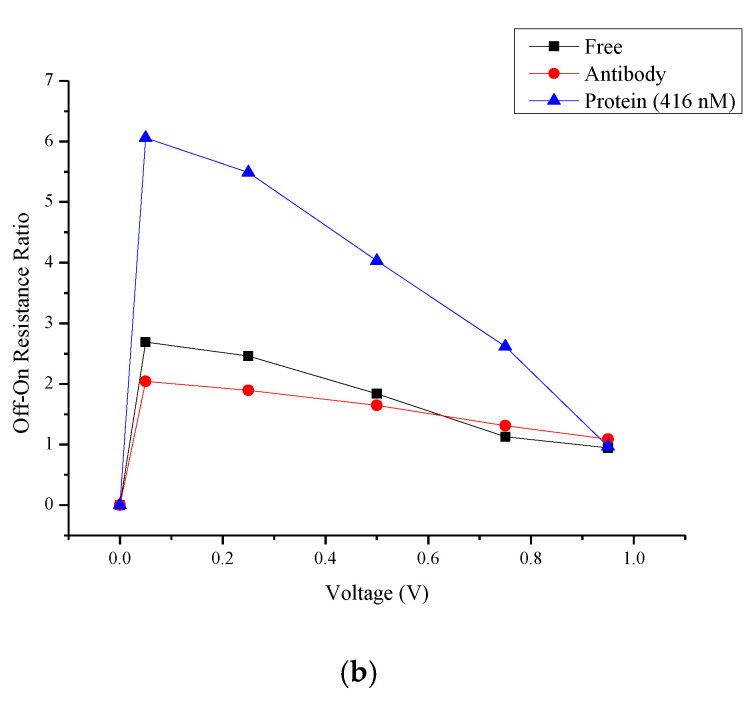
Characterisation plots; (**a**) *I-V* curve for 2 mm-diameter well; (**b**) Off-On resistance ratio of the memristor at 0 to 1V.

Figure 11 shows the characterization plots for the developed memristor sensor for the same three phases, as described/outlined in the methodology section. Aluminium contact and ITO are used as top, and bottom contact, respectively for *I-V* characterization. Aluminium is one of the best metal material that can provide good ohmic contact with TiO_2_ material [25]. The *I-V* curve for the representative sample (with 2 mm wells diameter and NS1 protein concentration of 416 nM) is shown in Figure 10a. It is observed that the largest loop size of the *I-V* curve was recorded for sensor under phase III (immobilised sensor with antibody + NS1 protein). Surface-free sensor (phase I) and antibody-conjugated sensor surface (phase II) recorded small changes in the loop size.

Based on Figure 11a, Off-On resistance ratio for the *I-V* curve for the representative sample is obtained and is shown in Figure 11b. Off-On resistance ratio was chosen as the mode of analysis to determine the sensor sensitivity due to its suitability to represent the loop size of the hysteresis loop, in general [14,26]. In general, larger loop size produces larger value of off-on resistance ratio and vice versa.

By computing the ratio, *R_off_/R_on_* and plotting against the concentration of NS1, it is observed that there was a significance change of the ratio values for phase III (with concentration of 416 nM), which is in agreement with the finding from Figure 11a. The changes confirm that the formation of the complex has changed the electrical properties, which is useful for immunosensing application. It indicates the validity of the chosen sensing technique for detecting the complex formation between antibody and antigen.

Table 1 shows the Off-On resistance ratio extracted from *I-V* curve for all well diameter dimensions (NS1 concentration of 416 nM), in order to investigate the effect of well diameter towards sensing performance.

In order Table 1 shows that the ratio is increasing as the diameter increased provided the reading is taken at the same reading voltage. Maximum value of resistance ratio that is obtained for the largest diameter agreed with the largest hysteresis loop, as depicted in Figure 10a.

The data collected for all experiments also shows a consistent trend of the ratio values with respect to diameter and NS1 concentration, which validates the sensing capabilities of the fluidic based memristor device developed in this work.

### 4.3. Sensitivity of Detection

**Figure 12:**
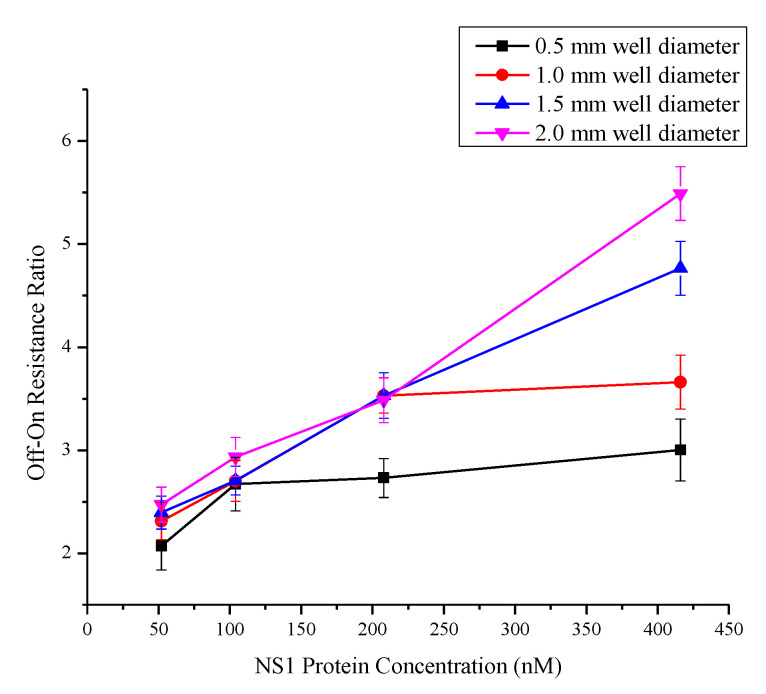
Off-on resistance ratio curve for four different wells diameter (0.5, 1, 1.5 and 2 mm) when applied with four concentrations NS1 protein (52, 104, 208 and 416 nM).

Figure 12 shows the overall performance of the immunosensing for selected concentration and well diameter of interests taken at 0.25V (reading voltage).

It is observed that for all diameter size, the detection can be achieved the for the maximum concentration 416 nM down to 52 nM [27]. However, at low concentration, the sensing capabilities (resistance ratio) is not clearly observed as compared to higher diameter sensing area. At less than 1mm of well diameter, the resistance ratio start to get nearly flat even at higher concentration. This is due the complex formation that get saturated at lower diameter of sensing the area. This suggests the importance of diameter to be taken into account. The best detection is observed at maximum diameter used, at 2mm. The measured ratio displays a good linear relationship, which is important for better resolution and sensitivity. In terms of sensitivity measurement, Figure 12 is used to obtain the sensitivity of the device, as outlined in the methodology section. Table 2 shows the sensitivity value calculated for the diameter of interest.

The table indicates that memristor with the well diameter of 2.0 mm recorded the highest sensitivity of 8.21 × 10^−3^ (nM)^−1^, indicating sensor with the largest diameter has the highest sensitivity towards the detection of NS1 protein. It is found that as the diameter increases, the surface area of TiO_2_ also increases, which results in the higher amount of immobilized NS1 antibodies. Upon the addition of the NS1 protein, the formation of the antibody-target complex is also elevated, which accounts for the increase of the Off-On resistance of the sensor surface.

## 5. Conclusions

In this study, fluidic-based memristor sensor for NS1 protein was successfully developed. The works proved the functionality of the proposed immunosensing mechanism. The developed sensor is viewed as one of the alternative for the label-free diagnostic of NS1 protein towards early detection of dengue in wet condition. The detection can be achieved for all the prepared concentrations of interests. For the concentration range of interest, the hysteresis loop and diameter of the sensing region becomes the contributing factor towards detection sensitivity and linearity. This work is significant in providing an alternative platform for future research work in fluidic based immunosensing application. This sensing mechanism can be further explored by focusing into areas, such as the material used due to the sensing dependency on resistance and hysteresis behaviour of immunoassay.

## Figures and Tables

**Table 1:** Off-On resistance ratio for different well diameter size.

Well-Diameter (mm)	Off-on Resistance Ratio
0.5	3.25032
1.0	3.84784
1.5	4.77501
2.0	6.05929

**Table 2:** Summary of third structure memristor sensor sensitivity for four wells diameters.

Wells Diameter (mm)	Sensitivity, S (nM)^−1^
0.5	2.06 × 10^−3^
1.0	3.58 × 10^−3^
1.5	6.57 × 10^−3^
2.0	8.21 × 10^−3^
